# The Jokes of Serious People

**Published:** 1892-01-02

**Authors:** 


### PASSING TOPICS.

### THE JOKES OF SERIOUS PEOPLE.

We have been lately told that the jokes of people ordinarily accounted serious must be received with suspicion. This appears decidedly hard upon those who are not indifferent to the charms of variety, and although we have no sort of intention to sacrifice willingly our own cherished character for strict sobriety, which we doubt not is well established, it is but prudent to provide for not impossible lapses.

Happily for themselves and the world, few men can be certain of retaining an unimpaired gravity. Some time or other it gives way and the sombre reflections of even the most serious among us are brightened by a sense of the ridiculous. It does not follow that a man's mind is allowed to lie fallow at this period; on the contrary, it is more than likely that, if he attempts to communicate his fun to other people, he only exchanges one field of labour for another. Thackeray tells us pathetically how difficult it is to "pump" jokes through several pages, and it is, without doubt, harder work to raise laughs than to prepare homilies. Like the Scotch laird, many of us "jock wi' deeficulty," while, in some rare instances, it is true, humour may be superabundant enough to call for caution in its exercise. A lineal descendant and heir of the fanciful creation of Oliver Wendall Holmes' verses who never dared to be as funny as he could, would be a decided acquisition and worth his weight in gold, upon occasions.

Seasons influence us a great deal, and we are all inclined to regard jokers as more or less privileged at Christmas time, for instance. We allow liberties then we might be quick to resent at a less favourable opportunity. It is remarkable how shrewdly the inveterate humourist reckons up the measure of our amiability, and taxes it accordingly. In wit, as in poetry, most of the professors are distinctly minor, and the greatest are, perhaps, the least conscious of their gifts. What could be more delightful than the naiveté of "One connected with many hospitals," who lately bespoke for hospital readers an *olla podrida* of odd volumes and waste newspapers, or the fine irony of the firm of bill stickers who have proposed to allow placards of charitable institutions to be affixed to the hoardings under their control free of charge during Christmas week? But for a masterpiece of seasonable frolic, nothing can compare with the "annual appeal" of the Charity Organization Society, whose aim it is, as we learn, "to draw people of all creeds and classes into a fellowship of charity, and of such charity as will stay and prevent distress and yet make the alms-seeker and the pauper to cease out of the land." Well, if the power of the society is equal to its aim, it ought to make a big hit, but surely its management is masquerading. That the affections of the society have ever been ardent enough to carry it beyond its own strict notions of propriety, none can affirm, but what are we to think of a programme which sets forth as an object of the society, the establishment of a Fellowship of Charity? It is a bold departure. To make the alms-seeker and the pauper to cease out of the land is a superhuman endeavour, and, without making a reflection, we shall prefer to appeal from Philip jocular, to Philip sober, in this matter. Whatever may be thought by other people, we may be certain that the alms-seeker and the pauper will have some doubt of the charitable character of an expatriation under such auspices.

AGRICOLA.

